# Supplementary material for: Are carbon emissions trading and green financial instruments synergistic? -Comprehensive quantitative research based on content analysis
Source: PLoS One. 2024 Mar 7;19(3):e0298601. doi: 10.1371/journal.pone.0298601 (PMC10919601; doi:10.1371/journal.pone.0298601)
Supplement: S1 Data — (ZIP) [file pone.0298601.s001.zip › Data/地级市Z_F工作报告.zip!/╦─┤¿/│╔╢╝╩╨/└┤╫╘╣1⁄2╓┌║┼í╛┬φ┐╦╩2╛▌═°í┐_2015.docx]

各位代表：

　　现在，我代表市人民政府向大会报告工作，请予审议，并请市政协委员和其他列席人员提出意见。

　　2014年工作回顾

　　2014年，面对国际环境错综复杂、国内经济下行压力加大的严峻形势，我们在党中央国务院、省委省政府和市委坚强领导下，在市人大市政协监督支持下，认真贯彻党中央国务院稳增长、促改革、调结构、惠民生一系列重大部署和省委省政府“三大发展战略”，深入实施“改革创新、转型升级”总体战略和五大兴市战略，及时出台了稳增长二十二条措施，坚定信心、克难奋进、主动作为，各项工作取得新的成绩，全市继续保持良好发展势头。预计全年地区生产总值迈上万亿元台阶，增长8.8%左右；实现固定资产投资6600亿元；一般公共预算收入1025亿元，增长14.1%；社会消费品零售总额4200亿元，增长12%；城镇居民人均可支配收入32665元，农民人均纯收入14478元，分别增长9%和11.5%。天府新区获批成为国家级新区。

　　一、经济保持平稳较快增长

　　一是工业经济快速增长。预计规模以上工业增加值增长12%以上。重点产业快速发展，突出发展电子信息、汽车、石化、轨道交通等产业，加快发展航空航天、生物医药、新能源、新材料、节能环保等产业，电子信息、汽车、机械产业主营业务收入分别达到2800亿元、1300亿元、1070亿元。重大项目加快建设，捷普生产基地、一汽铸造等重大项目建成投运，东风神龙乘用车等项目开工建设，超大型数据中心等项目进展顺利，438个工业重大项目完成投资620亿元，产值过百亿工业企业增至9家。加强节能减排，初步建立合同能源管理奖励全覆盖机制，全年淘汰落后产能企业55户。

　　二是现代服务业加快提升。预计实现服务业增加值5080亿元，增长8%。商贸物流、金融服务和总部经济等服务业核心功能进一步增强，国家电子商务示范城市建设取得新成绩，电子商务交易额突破5000亿元，“新三板”挂牌上市企业24家，荣获2014中国城市物流杰出成就奖。科技服务、信息服务、健康服务等新兴服务业加快发展，连锁经营、同城配送等新型业态发展迅速，会展节庆拉动消费超过580亿元，接待旅游总人数1.86亿人次，旅游总收入达1663.4亿元、增长25.1%。加快推进32个市级服务业集聚区建设，重大服务业项目完成投资1050亿元。

　　三是都市现代农业稳中提质。预计实现农业增加值360亿元，增长3.6%。深入实施“米袋子”“菜篮子”工程，新增粮食规模化经营面积17.9万亩，新建益民菜市（菜店）55家，粮食、蔬菜、肉类和水果等主要农产品产量稳定增长，全国都市现代农业暨“菜篮子”工程现场会在成都召开，并推广成都经验。特色优势产业发展壮大，粮经产业综合示范基地、现代农业精品园区、都市现代农业示范带加快建设，粮经复合种植模式和标准化生产全面推广，农产品精深加工产值突破900亿元。新建高标准农田27.8万亩，东风水库大坝枢纽工程基本建成。

　　四是自主创新能力切实增强。大力实施科技创新六大工程。编制完成轨道交通、生物医药、航空等产业技术路线图，参与制定2项国际标准和74项国家标准，企业累计建成各类研发机构738个。出台高校院所协同创新十条政策，推动校院地共建产业技术研究院、创新孵化载体和协同创新产业基地，中国工程物理研究院成都创新基地等科技成果产业化项目加快推进。设立债权融资风险资金池和创业天使投资引导资金，打造“科创通”创新创业服务平台，累计建成孵化器、孵化苗圃、大学科技园、科技楼宇等创新创业载体84家、在孵企业6500家。启动创新驱动发展试点示范区建设，成都高新区创建国家自主创新示范区取得积极进展。

　　二、新型城镇化建设有力推进

　　一是成都天府新区建设提速。编制完成直管区控制性详细规划，创新科技城、秦皇寺中央商务区和锦江生态带“一城一区一带”加快建设，新川创新科技园规划建设全面铺开。西部国际博览城、中铁西南总部等重大项目进展顺利，签约引进中建西南总部等重大项目129个。天府大道二期、剑南大道等骨干道路基本建成，天府新城污水处理厂、华阳东220KV变电站等基础配套项目加快建设。兴隆湖开始蓄水，鹿溪河生态区前期工作进展顺利。成都天府新区完成投资1383亿元。

　　二是城市基础功能明显提升。综合交通枢纽建设加力推进，成都新机场抓紧申报立项，成绵乐客专、成灌快铁彭州支线建成投运，五环路西段试通车，成渝客专、成兰铁路、成安渝高速等重大项目加快推进。市域快速通道路网更加完善，新邛快速路建成通车，城乡公交一体化稳步推进。缓堵保畅工程深入实施，城区骨干道路综合整治和重要节点改造加快，地铁3号线等5条线路7个项目建设全面推进，地铁2号线东延线开通运营，新开通免费社区巴士45条、定制公交15条，常规公交出行分担率提高2个百分点。成都第二水源李家岩水库前期工作取得重大进展，国家级互联网骨干直联点一期开通运行，率先在全国副省级城市实现村村通光纤，成为宽带中国示范城市和国家信息惠民试点城市。

　　三是城镇建设取得新进展。中心城区更新步伐加快，“北改”工程深入实施，棚户区、城中村和“三无”院落改造提速，“北改”项目建成投运67个，棚户区危旧房改造启动1.7万户。新型卫星城、区域中心城综合承载力增强，现代化小城市建设、特色镇打造和一般场镇改造步伐加快，26个镇入选全国重点镇。135个幸福美丽新村示范村启动建设。基本完成芦山“4•20”地震邛崃受灾乡镇农房维修加固和重建任务。基本划定市域城镇开发边界、工业集中发展区（点）范围，初步建立市域空间规划管制体系。

　　四是城乡人居环境不断改善。巩固全国文明城市建设成果，启动实施“四改六治理”十大行动。市容市貌管理切实加强，城市道路病害、违规户外广告、标识标牌等专项治理初见成效，生活垃圾处置设施加快完善。深入实施城乡环境治理，开展违法排污单位和百村容貌综合整治，实施大气污染防治行动计划，加强黑臭河渠综合治理，启动全国水生态文明试点城市建设，危险废弃物处置厂和新建污水处理厂一期建成投运，中心城区空气质量达标率上升24.9个百分点，率先在全国副省级城市实现乡镇（街道）环保机构全覆盖。切实加强城乡生态建设，划定市域生态保护红线和环城生态区生态用地边界，积极打造城镇生态隔离区，新增绿道216公里，香城、花田等5个环城生态区农田湿地建成开放，白鹤滩湿地公园被确定为国家级湿地公园，森林覆盖率提高到38.1%，获批成为国家生态文明先行示范区。

　　三、重点领域改革不断深化

　　一是经济体制改革稳步推进。启动实施商事登记制度改革，实行注册资本认缴登记制和企业、个体工商户年度报告制，积极推进电子营业执照，新登记企业增长56.5%。深化投融资体制改革，49项公共资源进入市公共资源交易中心集中交易，完善中小微企业投融资平台，民间投资占全市投资比重提高到57%。支持民营经济加快发展，落实小微企业税收优惠政策，扩大“营改增”试点范围，7家企业入选中国民营企业500强，民营经济占全市GDP比重提高到48.2%。

　　二是统筹城乡改革深入推进。农村产权制度改革进一步深化，支持农村集体经济组织利用集体建设用地发展工业、商贸物流和旅游业，稳妥推进农民住房抵押、担保、转让试点，各类农村产权抵押融资超过100亿元。新型农业经营体系不断完善，农业合作社、家庭农场、专业大户、龙头企业等新型农业经营主体新增3443家。全面推行农村小型公共基础设施村民自建，自建项目完成投资2.34亿元。统筹城乡综合配套改革示范建设深入开展，启动建设7个示范片。成功获批全国第二批农村改革试验区，农村产权制度改革荣获第七届中国地方政府创新奖。

　　三是社会治理改革有序推进。创新社区治理机制，推进信息化支撑、网格化管理、社会化服务，社区居委会实现全域直选，社区网格化管理被评为全国社会管理创新最佳案例。深化社会组织管理改革，支持社会组织参与公共服务和社会治理，扩大政府购买社会组织服务范围，鼓励开展公益志愿服务，全市社会组织增至8117个。推动社会治理重心下移，健全群众权益表达和协调机制。全面落实优抚安置政策，圆满完成征兵工作任务，军民融合深度发展。

　　四、对外开放水平加快提升

　　一是投资促进成效明显。预计实际到位内资3370亿元，实际利用外资100亿美元。抓住国际产业转移新趋势，用好财富论坛和华商大会后续效应，突出重点地区、重点产业和重点企业招商，签约引进英特尔骏马晶圆集成测试中心、北车成都轨道交通装备产业园、华侨城文化旅游等重大项目420个，落户世界500强企业增至262家。借力西博会、中外知名企业四川行等重大活动，依托新川创新科技园等平台，创新招商引资方式，提升投资促进实效。强化项目统筹协调和跟踪服务，健全企业诉求解决机制，被评为中国最佳营商环境城市。

　　二是外经外贸稳定增长。预计实现进出口总额3429.8亿元，增长9.3%，成都高新综合保税区进出口总额居全国第三、西部第一。加快以“全域通”、“区港联动”为支撑的关区通关一体化建设，进一步健全口岸服务体系，空港保税物流中心（B型）、青白江成都铁路临时对外开放口岸获批设立，外贸便利化水平明显提升。“蓉欧快铁”稳定开行，中亚列车开通运行，率先在中西部开通直飞美国客运航线，国际客货航线增至78条，双流国际机场年旅客吞吐量达3771万人次。企业走出去步伐加快，新增境外投资项目100个，对外承包工程和对外劳务合作营业额增长30%。

　　三是对外交流不断拓展。成功举办中德对话论坛等国际会议，国际政要来访增多，新增以色列和新西兰驻蓉总领馆，外国驻蓉领事机构增至12个。中新政府间合作新平台积极推进。设立国际友城留学生政府奖学金和公务员交流奖学金，国际友城和友好合作关系城市达61个，荣获国际友好城市交流合作奖。成功组织“2014伦敦成都周”、“铁人三项”世界杯等国际文体交流活动，城市国际影响力持续提升。

　　四是区域合作务实推进。主动发挥“首位城市”作用，认真落实成渝经济区成都城市群发展规划。推动成都经济区交通、通信基础设施互联互通，加快成都经济区区域协同创新，探索实施水污染、秸秆禁烧跨区域联防联控，实现成都经济区异地就医即时结算。与长三角、珠三角、环渤海等地区合作进一步加强。

　　五、城乡社会事业全面进步

　　一是就业社保体系加快完善。全市一般公共预算投入民生领域895亿元，174项为民办实事民生工程项目全面完成。健全就业服务体系，突出做好高校毕业生等重点群体就业创业，城镇新增就业24.3万人，失业人员再就业7.2万人，农村富余劳动力新增转移就业9.1万人，城镇登记失业率2.9%。完善城乡社保体系，城乡居民大病保险制度全面实施，城乡养老保险关系无缝衔接，6.3万名重度残障人、4680名低保对象纳入社会养老保险，最低月工资标准、低保标准分别提高16.7%、15.3%。竣工保障性住房2.3万套，发放住房公积金贷款106亿元。健全养老服务体系，推进居家、社区、机构养老综合发展，探索医养融合服务新模式，为14.3万名困难老人提供居家养老服务，建成城乡社区日间照料中心374个，新筹建养老床位9300张。成功创建全国残疾人文化建设示范市，连续三年蝉联新华社评选的中国最具幸福感城市第一名。

　　二是教育卫生服务更加优质。深入推进统筹城乡教育综合改革试验区建设，扩大普惠性学前教育资源总量，完成城乡中小学标准化提升工程，编制完成现代职业教育体系建设规划，实施优质教育资源远程推广计划，新增公办和公益性幼儿园74所，义务教育“择校热”有所缓解，教育发展指数位居全国副省级城市首位。深化医药卫生体制改革，推进全科医生执业方式和服务模式改革全国试点，县级公立医院全部取消药品加成，200家基层公益性医疗机构设备更新提升。全面实施“单独两孩”政策。成为全国促进流动人口社会融合示范试点城市。

　　三是公共文化服务明显提升。巩固国家公共文化服务体系示范区成果，积极开展“三下乡”、公益电影进影院等文化惠民活动，获批公共文化服务标准化建设、基层综合性文化服务中心建设国家级试点，被评为中国最具文化软实力城市。健全现代文化市场体系，推动文化创意、设计服务与相关产业融合发展，加快建设重大文化产业项目，成功举办2014成都创意设计周、第五届中国国际版权博览会和第二届中国网络视听大会，成都广告产业园获批国家级产业园区。深入开展全民健身活动，圆满完成第一届世界太极拳锦标赛承办任务，成功举办首届全民健身运动会，入围中国足球发展试点城市。

　　四是法治成都和平安成都建设扎实推进。加强社会治安管理，依法严厉打击违法犯罪行为，加强特殊人群和虚拟社会管理。严格食品药品和重要消费品安全监管，开展食用油、乳制品、调味品、中药材等重点领域专项整治，推动肉菜溯源系统“提质扩面”，实现食品药品从生产到消费全程统一监管。加快企业安全生产标准化和安全社区建设，开展道路交通安全综合整治攻坚年行动，强化油气管道、粉尘防爆、重大火灾隐患等专项整治，安全生产事故死亡人数下降10.4%。积极开展“法治大讲堂”、“法律七进”等普法活动，村社法律服务工作室实现全覆盖。积极回应群众信访诉求，初步建立逐级负责、链条管理的信访调处工作体系，依法预防和化解社会矛盾纠纷，社会继续保持安定和谐。

　　六、政府自身建设全面加强

　　一是依法行政深入推进。认真落实依法治市实施纲要，推进行政权力规范运行，强化重大行政决策合法性审查，完善行政执法自由裁量权基准制度，加强规范性文件备案审查和清理，组建了市级法律顾问团，农村产权纠纷仲裁工作荣获第三届中国法治政府奖。严格执行市人大及其常委会的各项决议决定，自觉接受市人大及其常委会的法律监督和工作监督。主动接受市政协的民主监督。办结人大代表、政协委员建议、提案1301件，办复率100%，满意和基本满意率99.85%。

　　二是行政效能有效提升。深入推进简政放权，动态清理行政审批事项，市级行政部门分别取消、调整行政许可27项、22项。政务中心服务水平进一步提高，市级集中审批整体授权率99.6%，现场办结和按时办结率均达100%。加快建设网上政务大厅和移动政务大厅，强化市长公开电话和“96960”行政效能投诉热线建设。深化政务信息公开，完善行政审批事项目录清单公开制度，强化财政资金使用、公共资源配置等重点领域的信息公开，健全政策文件日常解读人制度，政府信息依申请公开3000余件。

　　三是廉洁政府建设切实加强。扎实开展党的群众路线教育实践活动，持续聚焦“四风”问题深查实改，全市“三公”经费下降27.3%，市政府文件、全市性会议分别减少5.7%、13.3%。切实抓好中央巡视组反馈意见整改落实，认真开展“庸懒散浮拖”等五项专项整治，政府作风持续改善。落实党风廉政建设“两个责任”，着力健全惩治和预防腐败体系，加大审计监督力度，强化项目招标、政府采购、土地出让等重点领域监管，始终保持了惩治腐败的高压态势。

　　国家安全、防灾减灾、应急管理、消防、人防、保密、气象等工作不断加强，民族宗教、侨台、统计、档案、参事、地方志、移民等工作取得新成绩，妇联、工会、科协、残联、红十字会、哲学社会科学等工作取得新进步。

　　在此，需要特别说明的是，受国际环境错综复杂、国内经济下行压力加大，以及我国经济增长速度换挡期、结构调整阵痛期、前期刺激政策消化期“三期叠加”等多重因素影响，预计我市地区生产总值、社会消费品零售总额、城镇居民人均可支配收入等指标未能实现年初预期的目标。但经过全市上下的努力，全市经济社会仍然保持了良好发展态势，预计全年地区生产总值增速高于全国、全省平均水平。

　　各位代表，过去一年的成绩，来之不易，弥足珍贵。这是党中央国务院亲切关怀的结果，是省委省政府和市委坚强领导的结果，是市人大市政协和社会各界支持监督的结果，是全市人民共同努力的结果。在此，我代表市人民政府，向在各个领域和岗位上辛勤劳动、作出贡献的全市人民，向给予我们支持与监督的人大代表、政协委员、各民主党派、工商联和无党派人士、各人民团体、社会各界人士，向大力支持我们工作的中央和省驻蓉单位、解放军和武警驻蓉部队，向关心支持成都建设发展的港澳台同胞、海外华人华侨和国际友人，表示衷心感谢并致以崇高敬意！

　　在肯定成绩的同时，我们也清醒看到，当前我市经济社会发展还存在不少困难和挑战：宏观经济形势的严峻性、复杂性不容低估，企业扩产意愿下降，投资增速回落明显，工业提速乏力，新的消费热点带动不足，增长动能转换尚需时日，经济下行压力仍然较大；战略性新兴产业发展不够，产业结构需要进一步优化，创新驱动有待加强，经济转型升级的任务仍然艰巨；实体经济发展的要素瓶颈制约仍然存在，项目用地难、企业融资难融资贵等问题较为突出；交通拥堵、环境污染等“大城市病”未得到根本缓解，城市建设管理转型升级任务仍然艰巨；人民群众关心的一些热点难点问题有待进一步解决；一些重大改革虽已陆续展开，但重点领域和关键环节的改革突破还不够；有的政府工作人员改作风的自觉性和坚定性还不够强，“四风”问题在一些地方和部门仍不同程度存在。对此，我们将在今后工作中高度重视、科学谋划，不以事艰而退避，不以任重而不为，采取更有针对性、更有力的措施，切实加以解决，让全市人民看到实实在在的成效。

　　2015年工作安排

　　2015年，是实施“十二五”规划的收官之年，是改革发展任务艰巨繁重的一年。按照市委部署，做好今年政府工作要全面贯彻党的十八大、十八届三中、四中全会和省委十届三次、四次、五次全会精神，认真落实中央和省委经济工作会议部署，以邓小平理论、“三个代表”重要思想、科学发展观为指导，深入学习贯彻习近平总书记系列重要讲话精神，主动适应经济发展新常态，深入实施“改革创新、转型升级”总体战略和五大兴市战略，牢牢把握“稳中求进、统筹发展”工作主基调，坚持以提高发展质量和效益为中心推进经济建设，全面加强文化、社会和生态文明建设，持续保障和改善民生，推进城市治理体系和治理能力现代化，促进全市经济平稳健康发展、社会和谐稳定。

　　2015年全市经济社会发展主要目标是：地区生产总值增长8%左右，固定资产投资总额6650亿元，一般公共预算收入同口径增长8%，城镇居民人均可支配收入增长9%，农民人均纯收入增长10%，城镇登记失业率控制在4%以内，万元地区生产总值能耗进一步降低，主要污染物排放量进一步减少。我们要突破思维定势、转变思维方式，创新工作方法、狠抓工作落实，奋力完成今年发展目标和“十二五”规划确定的各项目标任务，同时编制好国民经济和社会发展“十三五”规划。重点抓好九个方面工作。

　　一、以稳中求进、统筹发展为基调，努力保持经济稳定增长

　　坚持同步提升经济规模总量和质量效益，更好发挥投资拉动增长的关键作用和消费拉动增长的基础作用，全面提升开放合作水平，推动经济可持续发展。

　　一是保持投资合理增长。投资是经济发展的底盘，抓好投资不仅能做强发展支撑，还能更好地促进消费。要坚持大抓项目、抓大项目，用好国家实施新一轮西部大开发、丝绸之路经济带和长江经济带战略等机遇，紧贴天府新区、“北改”、棚户区改造、交通、能源、水利、环保等重点领域，抓紧储备一批、签约一批、实施一批重大项目，进一步增强发展后劲、做强发展支撑。着力优化投资结构，发挥好财政投资在基础设施、民生事业等方面的引导作用，不断提升产业投资比重，加强技术改造、科技创新等领域的投资。全面增强社会投资活力，深化银企合作，健全以产业引导基金等方式为主的财政支持体系，积极开展PPP模式试点。实施重点项目760个以上、投资2100亿元以上。

　　二是积极扩大消费需求。充分发挥世界美食之都、中国最佳旅游城市、中国会展名城的品牌效应，大力挖掘旅游消费潜力，强化旅游公共服务设施建设，推动旅游与文化创意等产业融合发展，联合周边市州打造精品旅游景区、线路和产品，吸纳更多外部消费，加快建设世界旅游目的地和具有国际影响力的购物天堂。培育发展绿色消费、信息消费、文体消费、健康消费等消费热点，探索体验式消费等新型消费模式，促进个性化、多样化消费。增强居民消费能力，严格最低工资保障制度，多渠道增加城乡居民收入，改善消费预期、提振消费信心。进一步优化城乡市场环境，健全城乡市场流通体系，严格市场监管，优化市场秩序，加大消费维权力度，保持物价总水平基本稳定。社会消费品零售总额增长12%左右。

　　三是提升开放合作水平。营造“林秀鸟自来”的投资环境，拓展对外开放空间，提升全球资源整合能力，深度参与国际产业分工。提高招商引资专业化水平，用好重大投资促进活动、高端会展等平台，引进更多核心竞争力强、带动力大的国内外知名企业，实际利用外资超过70亿美元。推动高质量“走出去”，积极争创中国（成都）内陆自贸试验区，提升发展货物贸易和服务贸易，鼓励优势企业走出去开拓市场、参与国际竞争，推动成都产品、成都服务更广泛、更深入地走向世界。加强国际经贸合作，强化新川创新科技园、中法成都生态园等合作园区建设，依托“8+1”合作机制加强与芝加哥的商贸合作，积极争取中新政府间第三个合作项目。全面深化与长三角、珠三角、京津冀等地区的合作，完善成渝合作机制。强化“首位城市”作用，推进成都平原城市群协同发展。

　　二、以加快产业转型升级为取向，全面提升产业核心竞争力

　　产业是城市发展的根基，是推动成都科学发展、领先发展的支撑。要推动三次产业协调发展，优化产业结构，做强主业，完善配套，拉长产业链，形成产业集群，提高产业核心竞争力和话语权。

　　一是加快发展先进制造业。编制实施成都制造2025发展纲要，抓紧培育壮大千亿产业集群，提高高新技术产业占工业的比重，加快形成多点支撑、多元支撑的工业发展格局。率先发展信息安全、节能环保装备制造、页岩气、航空与燃机、新能源汽车等五大高端成长型产业，突出发展电子信息、轨道交通、汽车、石化等重点优势产业，大力发展航空航天、生物医药、新能源、新材料、节能环保等战略性新兴产业，培育发展北斗产业，推动冶金、建材、食品、轻工等传统产业向价值链中高端转移。推进一汽大众四期、京东方扩能、英特尔骏马、南车成都产业基地、新筑轨道交通装备制造基地等项目加快建设，确保一汽丰田2.7L普拉多、航宇航空发动机生产基地等项目竣工投产，完成工业投资1300亿元以上。深入实施大企业大集团培育工程、中小企业成长工程和微型企业扶持计划。加大节能减排力度，加快淘汰落后产能。规模以上工业增加值增长8.5%以上。

　　二是大力发展现代服务业。编制实施服务业2025发展规划，突出发展现代金融、现代物流、科技服务、电子商务、养老健康服务等五大新兴先导型服务业，加强要素市场建设，提高现代服务业占服务业的比重。加强资本市场和金融要素市场建设，大力引进各类金融机构，推动企业通过上市、债券等方式多渠道多层次融资，提升经济证券化水平。积极培育口岸经济和关税经济，争取设立青白江铁路保税物流中心（B）型，开通更多国际客货直飞航线，推动“蓉欧快铁”稳定双向开行，提升国际货运能力。加快阿里巴巴中国智能骨干网、万达云中心、京东西南运营中心、中建企业商务网、中药材天地网等重大项目建设发展。加快移动、联通、电信IDC和曙光成都云计算中心基地建设。大力发展会展、信息服务、软件和服务外包、文化创意、检验检测等现代服务业。推动传统服务业转型升级，强化春熙路、骡马市、建设路、新南天地等服务业聚集区建设，提升发展社区服务、农村服务等服务业。服务业增加值增长8.3%以上。

　　三是积极发展都市现代农业。深入推进“米袋子”“菜篮子”建设，严格落实耕地保护制度，稳定粮食产量，确保关系民生的主要农产品稳定安全供应。坚持用工业的理念发展现代农业，提高农业组织化、市场化、品牌化水平，扎实抓好粮经产业新村示范基地、现代农业精品园、都市现代农业示范带建设，积极发展现代种业、绿色有机农业、特色林业和精品养殖业，大力发展农产品精深加工业，打造更多农业“天府品牌”，让“天府粮”、“成都味”走进千家万户、走向全世界。全面落实强农惠农政策，推广先进适用技术，健全农产品流通和社会化服务体系，打造高素质职业农民队伍。深入推进农田水利建设，实施骨干渠系节水改造工程，加快山丘地区水源工程建设，完善高标准农田水利设施综合配套，增强农业可持续发展能力。

　　三、以经济体制改革为牵引，全面深化重点领域改革

　　坚持市场在资源配置中的决定性作用和更好发挥政府作用，按照“四化一驱动”改革要求，加强重点领域和关键环节改革，增强城市发展动力和活力。

　　一是着力深化经济体制改革。下大力气推动民营经济大发展大繁荣，坚持非禁即入放宽民营经济发展领域。深化商事登记制度改革和内贸流通体制改革，强化企业信用约束机制，落实中小微企业财税金融支持政策，激发社会创业与投资活力。加快推进国资国企改革，建立以管资本为主的国资监管体制，强化市属国有企业分类管理，稳步推进混合所有制经济发展，推动更多国有企业公众化、证券化。深化财税体制改革，加强专项资金和预算绩效管理，稳步推进“营改增”扩大试点。加快生态文明体制改革，健全生态补偿制度，推进排污权有偿使用和交易试点，加快建设节能量交易制度和交易平台，提高资源集约节约利用水平。

　　二是着力深化统筹城乡改革。加快全国第二批农村改革试验区建设，积极争取国家级试点，集成打造统筹城乡改革综合示范。深入推进农村产权制度改革，全面推开农业生产设施所有权、土地经营权登记颁证，实施农村集体资产股份化改革，在所有乡镇建成农村产权管理服务中心。加快构建新型农业经营体系，大力培育新型农业经营主体，创新农业适度规模经营方式，进一步放活土地经营权。深入推进城乡要素自由流动，支持开发新型涉农金融产品，推动农村产权规范流转交易。坚持农地农用，杜绝利用农地流转进行非农建设。深化户籍制度改革，探索建立外来人口积分落户办法，有序引导人口向中小城镇转移。

　　三是着力深化社会治理机制改革。发挥政府主导作用，鼓励社会各方面参与社会治理，推动政府治理和社会自我调节、居民自治良性互动。以增强社区功能、减少社区负担为重点，深入推进基层治理机制改革，进一步厘清基层自治组织职能，建立社区公共服务准入制度，鼓励农村小型公共设施村民自建，加强社会工作专业人才队伍建设，提升城乡社区自治能力。推进社会组织管理改革，支持社会组织依法加强自我约束、自我管理，鼓励发展志愿者队伍，更好发挥社会组织在公共服务和社会管理中的作用。加快推进中小城市综合改革试点，推广经济发达镇行政管理体制改革成果。深化事业单位分类改革。

　　四、以增强创新能力为核心，切实强化创新驱动发展

　　更多运用改革力量激发创新活力，聚集创新要素、汇聚创业力量，以高新区创建国家自主创新示范区为引领，努力打造创新驱动发展先行先试区，加快建设西部领先、国内一流的创新型城市。

　　一是增强自主创新能力。深入实施科技创新六大工程，编制实施创新型城市建设2025规划。支持成都高新区创建国家自主创新示范区，完善产业规划，优化空间布局，创新实施先行先试政策，打造创新驱动发展引擎；支持成都天府新区规划建设成都科学城；支持区（市）县开展创新驱动发展试点示范。强化企业创新主体地位，实施科技中小企业成长计划，支持大型企业建立联合研发中心。强化关键共性技术突破，完善重点产业技术路线图，力争在新一代信息技术、新材料、生物医药、轨道交通等重点领域，形成一批具有自主知识产权和核心竞争力的科技成果。支持企业加强标准研发，鼓励企业开展市场创新、管理创新、业态创新和商业模式创新。加快建设国家知识产权局专利审查协作中心，开工建设市质量技术监督检验检测服务中心。

　　二是完善科技成果转化机制。认真落实“成都十条”，强化校院地协同创新，支持在蓉高校和科研院所开展科技成果处置权、收益权和所有权改革，鼓励科技人才直接面向市场、直接进入产业体系和市场环节，支持科技人才创办领办企业。围绕主导优势产业发展，依托高校院所优质学科和专业，推动企校（院）政共建产业（技术）研究院、环高校特色知识经济圈和科技创新产业园，促进更多科技成果在蓉转化。统筹规划高校院所的科学研究、人才培养、社会服务等功能，推动高校与城市协同发展。

　　三是营造良好创业环境。启动实施“创业天府”计划，大力培育大学生创业先锋，壮大创新创业队伍，让“知识青年”转化为新型产业力量，形成万众创新、大众创业的局面。拓宽创业资本渠道，设立大学生创业天使投资基金，吸引国内外风投、创投机构聚集蓉城，支持创业和创新企业发展。深入实施成都人才计划，依托在蓉重点高校和国家级创新平台，引进更多高层次创新创业人才。深化“科创通”创新创业平台建设，大力发展创客空间、新型孵化器等创业载体，完善研发设计、技术交易、知识产权保护等科技服务，激活创新资源，增强创新发展的活力。

　　五、以再造产业成都为目标，加快建设成都天府新区

　　抓住天府新区成为国家级新区的机遇，以打造内陆开放经济高地、宜业宜商宜居城市、现代产业集聚区、统筹城乡一体化发展示范区为目标，加快建设国际化现代新城。

　　一是完善规划加快建设。完善新区发展规划，优化新区城市功能、道路交通、产业布局和生态建设体系。坚持以国际视野建百年工程，加快新区起步区建设，重点抓好“一城一区一带”、新川创新科技园、“一路一带”国际合作园建设。加强新区骨干路网建设，加快“三纵一横”二期、“三横一纵”工程建设步伐，推进天保大道、兴隆湖环湖路等道路和地铁1号线3期加快建设，构建以天府新城为中心、辐射周边的快速通道体系。健全区域协调推进机制，携手资阳、眉山片区联动发展、同步崛起。

　　二是加快现代产业集聚。高水平建设汽车产业功能区、空港经济开发区、现代制造业功能区、轨道交通产业园及新材料产业功能区等产业园区，大力发展现代物流、现代金融、总部经济等生产性服务业，配套发展生活性服务业，推动新区二三产业“两业并举”。加强统筹协调和要素保障，加快成都科学城、西部国际博览城等项目建设，确保保利国际等重大项目开工。强化招大引强，围绕电子信息、汽车、高端装备制造、新材料、现代服务业等重点领域，签约落户一批重大产业化项目。

　　三是着力完善综合服务功能。坚持功能复合、职住平衡，加快大源、中和等组团的商业、商务、交通和生活配套建设。完善公共服务和市政配套，加快新区学校、医院、变电站、消防站等公建配套和农民安置小区建设。提升新区生态环境质量，加快建设新区中央公园、天府新城污水处理厂，完成兴隆湖生态水环境综合治理、锦江生态带综合治理，打造滨水城市的靓丽景观。

　　六、以提升城市品质为抓手，提高城市现代化国际化水平

　　坚持一流标准，推动城乡基础设施规划建设互联互通，强化重点区域改造，加强“小街区规制”规划建设，增强城市综合承载能力，构建双核共兴、一城多市的网络城市群发展格局。

　　一是着力优化中心城区功能。深入实施“北改”工程，强化火车北站、八里庄等重点区域改造，健全医院、学校、菜市场等公建配套，加快建设畅通城北、宜人城北、发达城北。加快成都化工四厂、内燃机总厂生活区等棚户区和城中村改造，完善老旧院落安防、绿化等设施，改造中心城区老旧院落400个，完成远郊县市“三无”院落整治，调迁改造城区老旧市场14家以上。加强城市地下空间开发利用。提升城区无线局域网覆盖能力。力争开工建设李家岩水库、市自来水七厂二期。加强历史文化遗存遗迹保护，大力传承成都历史记忆，留住城市文化根脉和发展“年轮”，让人们记得住乡愁。

　　二是着力强化中小城镇建设。按照独立成市的要求，加快发展新型卫星城和区域中心城，优化规划、完善功能，培育壮大支柱产业，合理分工产业和功能。加快建设现代化小城市，强化产业支撑，提升综合服务和社会管理功能。加快特色镇建设和一般场镇改造，提升城镇内涵品质，因地制宜发展特色产业，增强辐射带动发展的能力，促进就地城镇化。统筹推进新农村综合体、新村聚居点和农村基础设施建设，加快产村一体发展，创建幸福美丽新村示范村100个以上。完成芦山“4•20”地震邛崃受灾乡镇灾后重建。

　　三是着力完善综合交通体系。坚持交通先行，加快构建内畅外通、区域辐射、联动世界的现代立体交通体系。做好成都新机场、成都新机场高速、成都经济区环线高速等重大项目前期工作，启动成彭高速扩能改造工程，推进成兰铁路、西成客专、川藏铁路、成蒲铁路、北站扩能改造等重大项目加快建设，力争成渝客专、成安渝高速、五环路建成投用。完善市域交通路网，推进城乡公交一体化，力争大件路外绕线、成温邛快速路、五洛路、沙西线快速改造工程建成通车。强化城区环线重要节点立交改造和射线通道建设，加快建设地铁3号线等5条线路，启动建设地铁6号线，开通运行地铁1号线南延线，开工建设有轨电车示范工程，大力发展大容量公交，进一步提高公交分担率，努力让市民出行更加方便。

　　四是着力营造国际化城市环境。提升国际化生产服务配套功能，加快区域通关一体化建设，发挥72小时过境免签政策效应。完善国际化生活服务配套，规划成都领馆区，引进更多国际学校、国际医院等涉外服务项目。加强多层次国际交流，争取设立更多驻蓉领事机构和国际组织分支机构，发挥国际友城、国际会议、国际合作项目等的影响和作用，加强对外宣传推介，提升城市国际知名度和美誉度。

　　七、以优化人居环境为重点，大力打造宜人成都

　　加快生态文明建设，加强城市综合管理制度建设，扎实推进“四改六治理”十大行动，坚持“天天归零”的理念，强化城市精细化管理，深入开展城乡环境综合治理，让天更蓝、地更绿、水更清。

　　一是加强城市综合管理。强化市容市貌治理，加强道路井盖、排水管网、架空管线等市政设施综合整治，强化户外广告招牌清理改造，扩大生活垃圾分类试点，加快生活垃圾处置设施建设，完善中心城区餐厨垃圾收运体系，完成120条中小街道路面“黑化”。加强交通环境治理，统筹重大城建项目施工，科学组织重要节点交通，完善智能交通信息系统，健全市域交通导识标志，推进停车泊位和停车换乘系统建设，扎实开展货车、出租车专项治理，严格施行成都市非机动车管理条例。加强违法违章建设治理，强化主要城市道路、交通枢纽、城市公园等区域违建整治，坚决杜绝新增违法建设。

　　二是加强城乡环境治理。下大力气治理大气雾霾，强化城乡快速通道路面、建筑工地扬尘整治，加大餐饮油烟整治力度，加强企业环境监管和污染治理，加快淘汰燃煤锅炉，推进秸秆综合利用和禁烧联防联控，落实烟花爆竹燃放管理规定，努力从源头上控制雾霾，改善全市空气环境质量。强化河渠污染治理，加快城区污水处理厂、污泥处置厂建设，完善城市地下管网收集系统，强化饮用水水源地保护，力争国控断面水质继续改善，实现水环境质量持续好转。加强农村环境治理，完善农村垃圾收运体系和处置设施，加大面源污染防治力度，提升农村人居环境质量。

　　三是加强城乡生态建设。坚持用生态的办法保护生态环境，提升中心城区绿地系统，加快城市公园、街头绿地建设，积极推广绿色建筑，中心城区新增公共绿地10万平方米。加快环城生态区建设，加强绕城生态带复耕植绿，强化河湖水系连通建设，构建环城湖泊湿地生态体系。严守市域生态保护红线，打造城镇生态隔离区，发展城市走廊间组团式绿地，加强龙门山、龙泉山生态屏障建设，抓好天保二期、野生动植物保护和退耕还林工程，争创国家生态市和全国水生态文明城市。

　　八、以增进民生福祉为追求，全面加快城乡社会事业发展

　　坚持守住底线、突出重点、完善制度，进一步提升公共服务水平，把发展成果更多转化为惠民实效，努力让全体市民过上更加幸福、更有尊严的生活。

　　一是健全就业社保体系。突出抓好高校毕业生、被征地农民等重点人群就业，着力解决就业结构性矛盾，推进职业技能素质提升工程，完善就业失业监测统计制度，动态消除零就业家庭。提升社会保障和救助水平，实施全民参保登记计划，深化医疗保险总额控制、付费方式改革，加强城乡养老保险市级统筹，做好机关事业单位养老保险改革前期工作，适时提高城乡居民养老保险待遇和低保标准，实现城乡低保标准分圈层统一。提升城乡住房保障水平，实行公共租赁住房和廉租房并轨运行，扩大住房公积金制度覆盖面，新建保障性住房4800套。健全城乡多元养老服务体系，建立经济困难老人基本养老服务补贴等制度，分层分类推进养老服务机构建设，提升养老服务标准化水平，新筹建养老床位10000张，让老人干干净净活出尊严、舒舒服服活出品质。

　　二是提升公共服务质量。深化统筹城乡教育综合改革试验区建设，推动义务教育优质均衡发展，加快发展现代职业教育和社区教育，高水平办好成都大学，服务好在蓉高校发展，建成40所教育国际化窗口学校，力争3年内每个街道、每个乡镇都有一所二级以上公办或公益性幼儿园，今年新（扩）建公办幼儿园150所。完善现代公共文化服务体系，深入推进文化惠民工程，加快基层综合性文化服务中心建设，深化公益性文化事业单位改革，规划利用好“非遗”场馆，推动“非遗”项目生产性传承，办好第五届国际“非遗”节和成都文化创意设计周，建成开放成都博物馆。深化医药卫生体制改革，加快基层卫生综合改革，强化医疗机构药品集中采购，加强妇幼健康服务，健全计生特殊困难家庭长效扶助机制。广泛开展全民健身活动，推动区（市）县“一场一馆一池”、“两个中心”建设，实现村（社区）体育健身设施满覆盖。办好全国第九届残运会暨第六届特奥会。扎实做好民族宗教工作。

　　三是深化平安成都建设。提升社会治安水平，健全立体化社会治安综合防控体系，依法严厉打击严重暴力、网络犯罪、非法集资和“黄赌毒”等违法犯罪活动，完善反恐防暴预警应急机制，不断提高市民的安全感。提升食品药品安全监管水平，深入推进溯源管理和市场准入，严厉打击食品药品违法犯罪行为，构建食品药品社会多元共治机制，启动市食品药品安全检验检测研究院提档升级工程。强化突发事件预测、预警和预防。提升信访工作法治化水平。做好国防动员工作，启动创建新一届全国双拥模范城。严格安全生产责任，建立隐患排查治理动态监管体系，强化道路交通、人员密集场所、建筑工地、危化品、特种设备等重点领域安全监管，坚决杜绝重特大安全事故，切实保障城市安全、市民安宁。

　　此外，今年我们还将为市民办“十件实事”，包括城市改造、环境治理、扶贫解困、就业促进、教育助学、医疗卫生、社会保障、百姓安居、文化体育、基础设施建设等方面。

　　九、以提高城市治理能力为导向，强化政府自身建设

　　经济社会加速转型，要求城市治理方式随之转变。必须坚持以人为本、民生优先、法治为基的现代城市治理核心理念，强化政府自身建设，提升城市治理体系和治理能力现代化水平。

　　一是建设法治政府，提升依法治理水平。法治是现代城市治理的基石。要大力推进依法治市，强化政府依法行政、企业合法经营、市民守法自律，营造全民尊法信法守法用法的良好氛围。严格执行市人大决议决定，自觉接受市人大及其常委会的法律监督和工作监督，认真办理人大代表建议。主动接受市政协的民主监督，主动听取市政协的意见，认真办理政协提案。广泛听取民主党派、工商联、无党派人士和工会、共青团、妇联等人民团体意见，高度重视社会监督和新闻舆论监督。加快建立权力清单、责任清单、负面清单和程序清单，完善重大行政决策法定程序，建立重大决策终身追究制和责任倒查制，健全行政执法自由裁量权基准制，坚持严格规范公正文明执法。深入实施“六五”普法，大力开展“法律七进”活动。

　　二是建设效能政府，提升政务服务能力。大力推进简政放权，以进一步减程序、减费用、减时限为重点，深入推进行政审批制度改革，全面清理和规范行政许可事项，取消全部非行政许可审批事项，大幅减少投资项目前置审批，消除行政审批“灰色地带”。全面推进政务公开，加大财政预算、重大建设项目、公共资源配置、公益事业建设等重点领域信息公开，做到决策公开、管理公开、服务公开、结果公开，确保权力在阳光下运行。加强电子政务建设，拓展政务大厅功能，推进全程网上审批，让市民少跑路、少受累。加强智慧城市建设，加快建立城市治理信息系统，统筹推进感知基础设施、网络基础设施、云计算基础设施、地理空间基础设施建设，促进城市信息的高效传递和智能响应，为治理城市提供及时、准确、科学的服务，降低治理成本、提高治理效率。

　　三是建设廉洁政府，提升政府公信力。落实“三严三实”要求，落实党风廉政建设“两个责任”，落实党的群众路线教育实践活动和巡视反馈问题的整改措施，严防“四风”问题回潮反弹，推进政风进一步好转。坚持抓改革发展与反腐倡廉“两轮同轴、力度统一”，健全惩治和预防腐败体系，加大财政资金分配、重大项目审批、重大物资采购招标等重点领域审计力度，强化公共资源交易监管，推进廉洁成都建设。完善债务管控机制，强化“三公”经费管理，稳妥推进公务用车制度改革，把更多的财力用于保障和改善民生。坚持科学理政、团结共事，将真抓实干作为政府工作的座右铭，打造守规矩、有作为的公务员队伍，努力以政府的勤奋指数，提升市民的幸福指数、城市的繁荣指数。

　　各位代表，今天的成都，已站在“新常态、万亿级”的新起点，踏上了创新驱动、转型发展的新征程。让我们紧密团结在以习近平同志为总书记的党中央周围，在省委省政府和市委坚强领导下，紧紧依靠全市人民，团结一心、坚定信心、开拓创新，推动全市经济社会发展再上新台阶，为成都打造西部经济核心增长极、加快建设现代化国际化大都市而努力奋斗！
